# Model Catalysis with HOPG-Supported Pd Nanoparticles and Pd Foil: XPS, STM and C_2_H_4_ Hydrogenation

**DOI:** 10.1007/s10562-021-03868-2

**Published:** 2021-12-06

**Authors:** Md. Abdul Motin, Andreas Steiger-Thirsfeld, Michael Stöger-Pollach, Günther Rupprechter

**Affiliations:** 1 Institute of Materials Chemistry, Technische Universität Wien, Getreidemarkt 9/BC/01, 1060 Vienna, Austria; 2 University Service Center for Transmission Electron Microscopy (USTEM), Technische Universität Wien, Wiedner Hauptstraße 8-10, 1040 Vienna, Austria; 3 Present Address: Department of Chemistry, University of California, Riverside, 501 Big Springs Road, Chemical Sciences #141, Riverside, CA 92521 USA

**Keywords:** Palladium, Platinum, Carbon, Model catalysts, Ethylene hydrogenation, Photoelectron spectroscopy, Scanning tunneling microscopy, Kinetics, Metal-support interaction

## Abstract

A surface science based approach was applied to model carbon supported Pd nanoparticle catalysts. Employing physical vapour deposition of Pd on sputtered surfaces of highly oriented pyrolytic graphite (HOPG), model catalysts were prepared that are well-suited for characterization by X-ray photoelectron spectroscopy (XPS) and scanning tunneling microscopy (STM). Analysis of the HOPG substrate before and after ion-bombardment, and of Pd/HOPG before and after annealing, revealed the number of “nominal” HOPG defects (~ 10^14^ cm^−2^) as well as the nucleation density (~ 10^12^ cm^−2^) and structural characteristics of the Pd nanoparticles (mean size/height/distribution). Two model systems were stabilized by UHV annealing to 300 °C, with mean Pd particles sizes of 4.3 and 6.8 nm and size/height aspect ratio up to ~ 10. A UHV-compatible flow microreactor and gas chromatography were used to determine the catalytic performance of Pd/HOPG in ethylene (C_2_H_4_) hydrogenation up to 150 °C under atmospheric pressure, yielding temperature-dependent conversion values, turnover frequencies (TOFs) and activation energies. The performance of Pd nanocatalysts is compared to that of polycrystalline Pd foil and contrasted to Pt/HOPG and Pt foil, pointing to a beneficial effect of the metal/carbon phase boundary, reflected by up to 10 kJ mol^−1^ lower activation energies for supported nanoparticles.

**Graphical Abstract:**

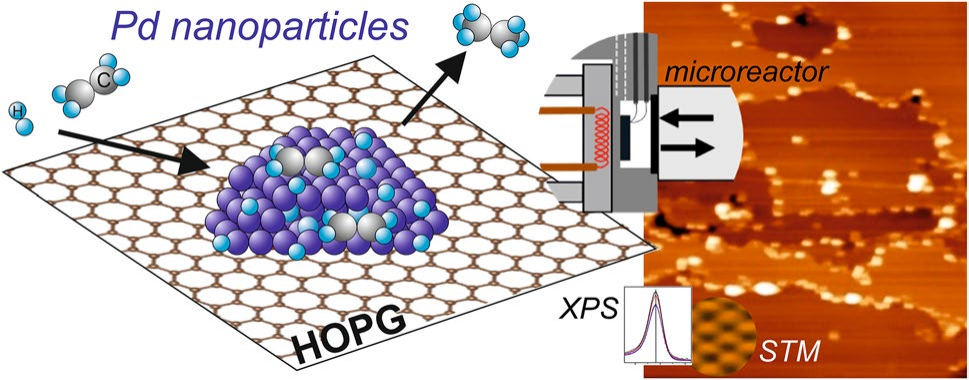

## Introduction

Supported metal nanoparticles are used in many fields of catalysis (environmental, chemical synthesis, energy generation by fuel cells, etc.), with Pt and Pd being specifically important. With respect to hydrogenation reactions, supported Pd nanoparticles are known to be the most selective, avoiding dehydrogenation and deactivation by undesired carbonaceous species. Accordingly, (selective) hydrogenation reactions have been thoroughly studied, e.g., of ethylene [1–7], 1-butene [8, 9], 2-butene [10], 1,3-butadiene [11–18], acetylene [19–21], propyne [22, 23] or unsaturated aldehydes [24, 25]. For reviews and further references refer to [26–35].

Especially when using carbon as support material [36], Pd nanoparticles exhibit favorable catalytic performance in selective (de)hydrogenation [37–41], oxidation [42, 43] and coupling [44]. Despite their nominally simple composition, carbon supports may exhibit complexity in terms of morphology (activated carbon powders, nanotubes, graphene layers and nanoplatelets) and functionalization (e.g., doping by N or B [45]), enabling fine-tuning of applications in sensing. thermal- and electro-catalysis [46–54]. Furthermore, the carbon support may directly affect the Pd nanoparticles by formation of subsurface carbon or a metal–carbon phase [30, 55–58]. Carbon deposits on metal surfaces have been reported to modify (de)hydrogenation [23, 34, 59–62], presumably by selective site blocking. Electronic interactions at metal/carbon interfaces can alter the binding energy of adsorbates [48, 49, 63] and potentially enhance the hydrogen availability [3, 30, 35, 47, 63–67].

In the current study, we employ a surface science model catalyst approach by growing Pd nanoparticles in ultrahigh vacuum (UHV) on a single crystal substrate of highly oriented pyrolytic graphite (HOPG). Alike studies on model oxide supports [4, 32–34, 68–77], this yields impurity-free samples that are amenable to many surface-sensitive techniques [78]. HOPG supports have been previously used for studies of thermal- and electrocatalysis on Ag, Pt, Pd and alloy nanoparticles [79–85]. In a number of studies, graphene grown on Ir(111) has also been used as model carbon support for Ir [86, 87], Pd [88, 89], Pt [90–94], Na [95], Sm [96], W and Re clusters [97].

Herein, two UHV-grown Pd/HOPG model catalysts were characterized in situ by X-ray photoelectron spectroscopy (XPS) and scanning tunneling microscopy (STM), indicating mean Pd particle sizes of 4.3 and 6.8 nm. Their catalytic activity in ethylene hydrogenation, a prototype reaction [2–7, 98, 99], was examined in a flow microreactor at atmospheric pressure, with gas chromatography (GC) and differentially-pumped mass spectrometer (MS) reactant/product analysis. Polycrystalline Pd foil was employed for comparison, characterized ex situ by scanning electron microscopy (SEM), electron backscatter diffraction (EBSD) and energy-dispersive X-ray fluorescence (EDX). The results are further contrasted to those of analogous Pt/HOPG model catalysts, reported by some of us earlier ([85]; adapted in light of the current GC analysis), and extended by additional measurements on polycrystalline Pt foil. Altogether, this confirms the feasibility of the current surface science approach to model carbon supported Pd and Pt catalysts, but specifically points to a prominent role of the metal/carbon phase boundary, an effect recently reported for Pd nanoparticles on graphene nanoplatelets and attributed to interfacial hydrogen [47]. The current study is a first step toward more complex reactions, such as selective olefine/diene [9–17] or alkyne [19–23] hydrogenation, on more complex bimetallic nanoparticles [100–102], bridging both the materials and pressure gaps [4, 73, 74, 101].

## Experimental

### Preparation of UHV-Grown Pd/HOPG Model Catalysts

**Fig. 1:**
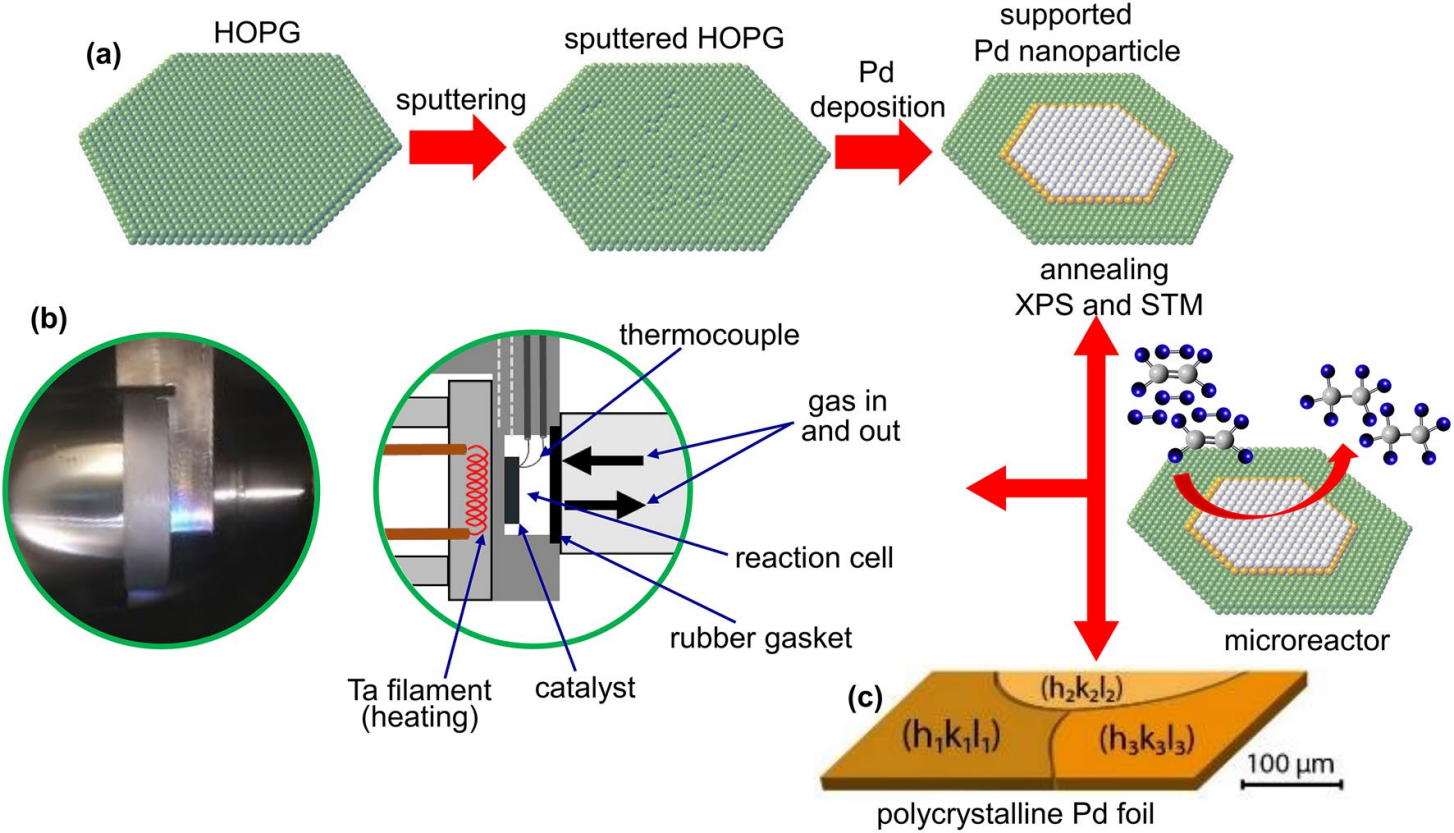
**a** Scheme of the UHV-preparation of Pd/HOPG model catalysts, including steps of HOPG “soft” sputtering, Pd deposition and annealing. In situ characterization is carried out by XPS and STM. The flat “raft-like” shape of the Pd particles was inspired by the high size/height aspect ratio revealed by STM (see below). **b** Catalyst samples are then mounted in a microreactor compartment, which together with an oven for external heating (left; also forming a recess for the reactor) and a gas supply tube (right; sealed via a Kalrez O-ring and mounted on a heavy-duty linear transfer) forms the flow microreactor assembly. **c** Polycrystalline Pd foil serves as a reference

Figure 1 shows a schematic overview of the preparation of the HOPG supported Pd nanoparticles, their characterization and testing in a flow microreactor under atmospheric pressure. The preparation is carried out in a UHV chamber, which is equipped with XPS (SPECS XR 50, with Al K_α_ and Mg K_α_ anode; EA 100 PHOIBOS) and connected to a second UHV chamber for STM (SPECS STM 150 Aarhus). The chambers are linked via magnetically coupled sample transfer rods and a load-lock enables rapid sample loading/unloading.

The preparation of Pd/HOPG followed procedures already used for Pt/HOPG [85], Ag/HOPG [79], PdAu/HOPG [81, 82] and Pd/ or PtGa/HOPG [83, 84]. Prior to installation in UHV, to create a fresh smooth surface, the upper layers of the HOPG crystal (7 × 7 × 0.8 mm, from NT-MDT, ZYA, 0.3–0.5° mosaic spread) were removed by adhesive scotch-tape. In UHV, the HOPG surface was annealed at 700 °C (60 min), followed by “smooth Argon ion etching” (Fig. 1a; SPECS IQE 35 sputter gun; 2 × 10^−6^ mbar Ar, 0.5 kV; few seconds). This has been shown to be a prerequisite to grow well-dispersed and stable (“anchored”) nanoparticles [79, 81–85, 103–106]. Using STM, atomic force microscopy (AFM) and infrared reflection absorption spectroscopy (IRAS with CO as probe molecule), Kettner et al. [83] demonstrated that prisine HOPG exhibited large, nearly defect-free terraces (up to micrometers) separated by “monoatomic” steps, whereas Ar^+^ bombardment created additional multilayer steps (up to 4 nm in height) and strongly disrupted the surface (“scattered graphene flakes ~ 10 nm in size with up to 2.5 nm thickness”).

Pd nanoparticles were then grown in UHV on the sputtered HOPG surface at room temperature (on a circular area of 0.38 cm^2^) by physical vapor deposition (PVD) of a Pd rod (Fig. 1a; Omicron EFM 3 T electron-beam evaporator with internal flux monitor). While the nucleation density (particles/cm^2^) was controlled by the substrate sputtering described above, the size of the Pd nanoparticles was adjusted by the amount of Pd deposited [107], expressed below as “nominal thickness”, i.e., monolayers (ML) of uniform Pd film. The number of deposited Pd atoms was determined by XPS and also independently calculated from STM analysis. After Pd particle growth, the model catalysts were heated to 300 °C (60 min) in UHV, which is about twice the highest catalytic reaction temperature applied herein. Documented by the pre- and post-treatment/reaction XPS and STM characterization of HOPG-supported (bi)metal nanoparticles by A.V. Bukhtiyarov et al. [81, 82], air exposure, repeated UHV annealing at 300 °C, and even reactive/oxidative treatments up to 250 °C/200 mbar did not change the mean nanoparticle size or distribution. The model catalysts remained stable and only for bimetallics surface segregation may occur, which is not relevant herein. Based on CO-IRAS, Libuda and coworkers [83] also reported that anchored Pd nanoparticles were stable and did not undergo strong sintering even upon annealing to 275 °C in 10^–6^ mbar CO or O_2_, just some faceting occurred. Altogether, this ensures thermally stable Pd particles with no sintering being expected during reaction.

Polycrystalline Pd foil (Fig. 1c; ~ 1 cm^2^; from Goodfellow) was used as a reference catalyst. It was cleaned in UHV by sputter/annealing/oxidation/reduction cycles until its front side was “XPS-clean”, assuming an average Pd density of 1.45 × 10^15^ atoms cm^−2^ [108]. The same procedure was applied to Pt foil of the same size.

### Characterization and Kinetic Tests of Pd-Based Model Catalysts

Ex situ transmission electron diffraction (TED) was used to analyze a thin HOPG film transferred via scotch-tape to a sample holder. The prepared model catalysts were characterized in situ by XPS and STM to determine the amount of deposited Pd and the nucleation density (number of particles/cm^2^), respectively, with both combined to calculate the mean size of the Pd nanoparticles. STM also yielded an apparent particle size. Polycrystalline Pd and Pt foils were characterized ex situ by SEM, EBSD [109, 110] and EDX, as well as in situ by XPS.

After characterization, each model catalyst was transferred to another smaller UHV chamber connected to a flow microreactor setup (Fig. 1b), described in detail previously [108]. A model catalyst was first attached to a stainless-steel sample holder inside a microreactor (volume ~ 4 ml) (Fig. 1b; middle) with temperature readout by a Ni/Ni–Cr thermocouple. The microreactor cell with the model catalyst can then be moved to a lower section of the UHV chamber, where it connects to a stainless-steel anvil (Fig. 1b, left) that also houses a Ta filament for heating the backside wall of the microreactor. The reaction compartment is then sealed from the other side by a movable gas supply tube (including gas inlet and outlet and actuated by a heavy-duty linear motion), which has a Kalrez O-ring at its front for sealing off the reactor (Fig. 1b, middle). The gas feed is adjusted by mass flow controllers (MKS) and the exit gas composition is analyzed by a differentially-pumped mass spectrometer (Hiden HPR 20) and quantified by gas chromatography (Micro GC Fusion, INFICON). High purity (5.0) gasses supplied by Messer Austria were used in all experiments. The maximum temperature of the reactor is 320 °C, due to the temperature limit of Kalrez 7075. As a blind test, the catalytic activity of the empty microreactor in ethylene hydrogenation was measured [108], showing a conversion < 5% at 100 °C. Accordingly, the evaluation of catalytic data was typically restricted to below 100 °C herein.

## Results and Discussion

### Characterization of the HOPG Substrate

**Fig. 2:**
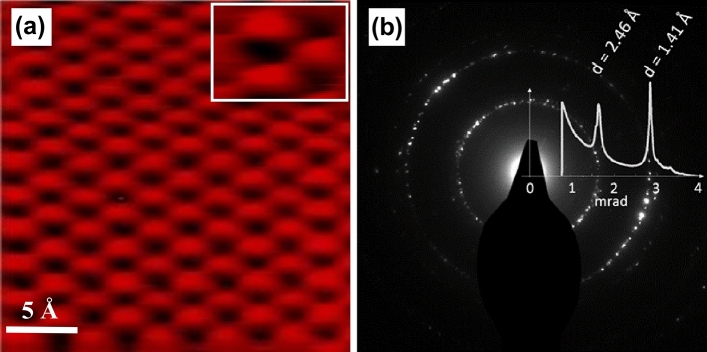
HOPG model support at 300 K: **a** STM image (V_bias_ = 0.4 V, I = 0.6 nA) with higher magnification inset and **b** TED pattern recorded at 200 keV with an intensity line profile as overlay

**Fig. 3:**
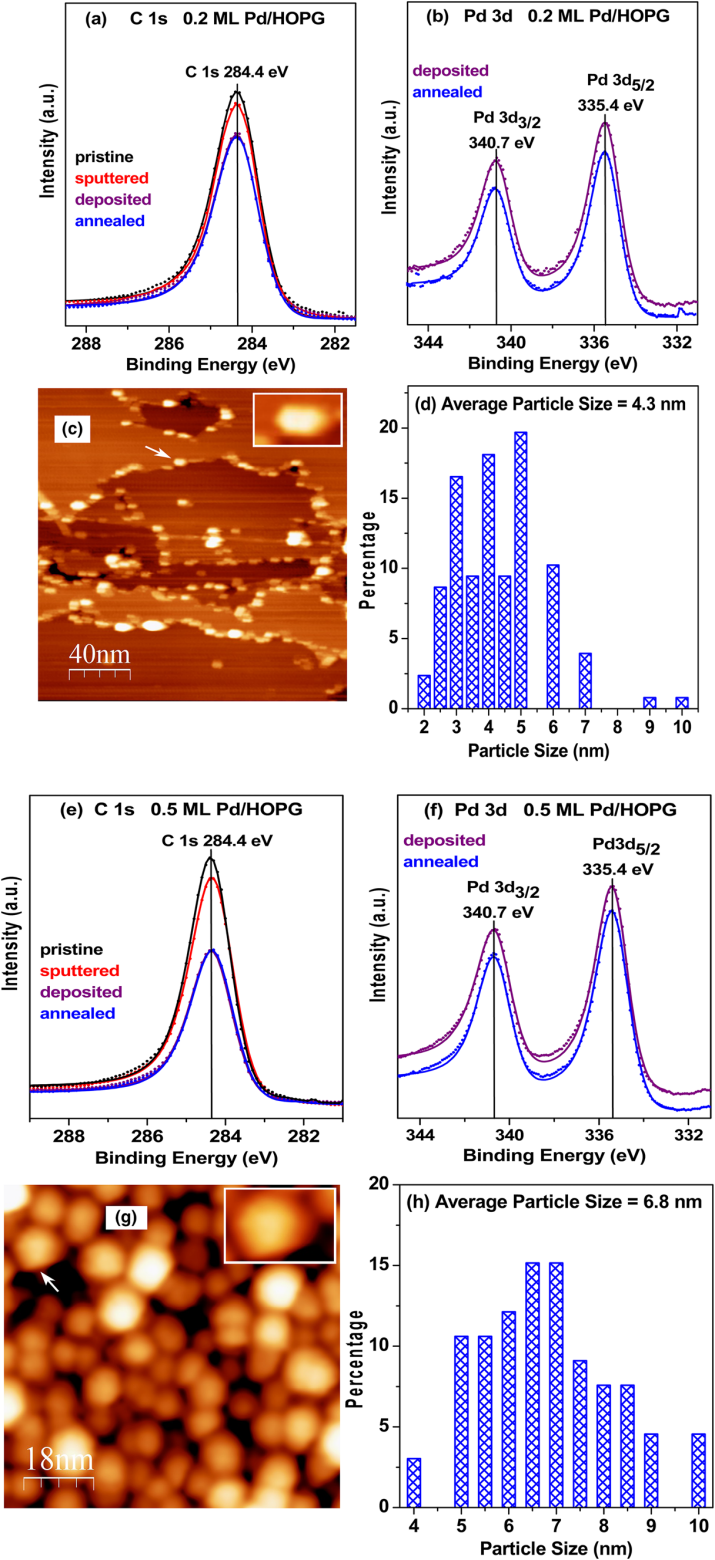
XPS spectra, STM images and size histograms of 0.2 ML Pd/HOPG (**a–d**) and 0.5 ML Pd/HOPG (**e**–**h**): (**a**, **e**), C1s spectra of the pristine HOPG surface (black), after sputtering (red), after Pd deposition (purple), and after annealing (blue) in UHV to 300 °C (1 h). **b**, **f** The corresponding Pd 3d spectra obtained after Pd deposition and UHV annealing at 300 °C. **c**, **g** STM acquired after annealing to 300 °C. The insets show higher magnification of the marked particles. Tunneling parameters: 0.47 V, 0.88 nA. **d**, **h** particle size histograms

The freshly “cleaved” HOPG surface (3.8 × 10^15^ atoms cm^−2^) was characterized by STM (Fig. 2a) [85] and transmission electron diffraction (TED, Fig. 2b), both confirming the expected crystallinity and surface structure of the substrate. XPS spectra of HOPG are included in Fig. 3, indicating a clean surface without impurities.

### Characterization of Pd-Based Catalysts

Previous studies had indicated that nanoparticles grown on pristine HOPG would be comparably larger and more prone to sintering than those grown on sputtered HOPG [79–85]. Consequently, ion-bombarded HOPG was employed as a substrate (Fig. 1a). As reported previously [85, 111] and stated above, soft argon ion etching of a carbon surface leads to surface disorder [79, 83, 112, 113] and Ar implantation. As in our previous study on Pt/HOPG [85], the number of removed/displaced carbon atoms was calculated from a quantitative analysis of the XPS C1s spectra before and after sputtering (Figs. 3a, c, e). After ~ 6 and ~ 9 s sputter times, the reduced C1s intensities indicate “nominal” defect densities of 2.00 × 10^14^ and 2.95 × 10^14^ defects/cm^2^ (Table 1; subsequently used for growing 0.2 ML Pd/HOPG and 0.5 ML Pd/HOPG samples, respectively). As mentioned, this treatment creates more nucleation centers for metal growth, better anchoring and stabilizing the resulting nanoparticles (note, however, that the resulting particle density reported below is still ~ 1000-times lower than the “nominal” defect density).

**Table 1:** Structure and reactivity data of Pd/HOPG and Pd foil model catalysts. Pt/HOPG and Pt foil are included for comparison

Model catalysts	0.2 ML Pd/HOPG	0.5 ML Pd/HOPG	Pd foil	0.31 ML Pt/ HOPG	0.74 ML Pt/ HOPG	Pt foil
HOPG defect density; from XPS (defects/cm^2^)	2.00× 10^14^	2.95 × 10^14^	n.a	2.32 × 10^14^	6.62 × 10^14^	n.a
Total number of metal atoms; from XPS (atoms/cm^2^)	3.06 × 10^14^	7.65 × 10^14^	n.a	4.66 × 10^14^	1.12 × 10^15^	n.a
Particle number density; from STM (particles/cm^2^)	7.94 × 10^11^	9.42 × 10^11^	n.a	6.14 × 10^12^	3.02 × 10^12^	n.a
Number of metal atoms per particle; from XPS/STM (atoms/particle)	387	812	n.a	76	371	n.a
Mean particle diameter; from STM (nm)	4.3	6.8	n.a	2.0	3.6	n.a
Mean particle height; from STM (nm)	0.5	0.6	n.a	0.4	0.6	n.a
Number of metal atoms per particle; from STM (hemispherical cap) (atoms/particle)	292	802	n.a	40	206	n.a
Number of metal surface atoms per particle; from STM (hemispherical cap) (atoms/particle)	214	518	n.a	33	89	n.a
Ratio of metal perimeter atoms to surface atoms; from STM	0.26	0.17	n.a	0.60	0.35	n.a
Total number of metal atoms; from STM (atoms/cm^2^)	2.32 × 10^14^	7.56 × 10^14^	n.a	2.46 × 10^14^	6.22 × 10^14^	n.a
Total number of metal surface atoms in the sample; from STM for particles (atoms)	6.46 × 10^13^	1.85 × 10^14^	1.45 × 10^15^ (front side)	7.75 × 10^13^	1.05 × 10^14^	1.45 × 10^15^ (front side)
TOF (s^−1^)	3895 (95 °C)	1478 (95 °C)	190 (95 °C)	284 (100 °C)	377 (100 °C)	204 (100 °C)
TOF (s^−1^)	2249 (65 °C)	871 (65 °C)	92 (55 °C)	168 (60 °C)	250 (60 °C)	49 (60 °C)
E_a_ (kJ/mol)	28.6 ± 2.2	27.9 ± 2.2	34.2 ± 2.5	27.3 ± 3.0	25.4 ± 1.4	35.5 ± 6.0

In the next step, Pd nanoparticles were grown on sputtered HOPG by PVD, resulting from nucleation and growth processes [74, 107]. The exact Pd amount deposited on HOPG was determined in situ by quantitative analysis of the XPS C 1 s intensity, which was reduced upon Pd deposition, by Pd 3d spectra, and by STM imaging (see below).

Figure 3a shows C1s spectra, and Fig. 3b the corresponding Pd 3d XPS spectra of the lower-loading Pd/HOPG model catalyst. The nominal Pd thickness was determined by comparing the peak intensity of C 1 s of sputtered HOPG with that after deposition/annealing at 300 °C. Utilizing the intensity analysis described in [114] and assuming homogeneous 2d-Pd layers and an implanted Ar monolayer underneath (inelastic mean free path of Pd and Ar of 1.6 and 2.973 nm, respectively), comparing the C1s peak area before Pd deposition and after deposition/annealing indicated a nominal Pd thickness of 0.2 ML (with 1 ML referring to 1.53 × 10 ^15^ Pd atoms cm^−2^).

Figure 3e shows C1s spectra of the second model catalyst, including pristine HOPG, after sputtering, after depositing a larger amount of Pd, and after annealing to 300 °C, with Fig. 3f displaying the corresponding Pd 3d XPS spectra. The nominal thickness of this Pd overlayer was calculated analogously to be 0.5 ML.

It is important to note that for both samples, the intensity of the C 1 s photoelectrons of the HOPG substrate was attenuated after Pd deposition, but that annealing did not lead to a further intensity reduction (or only ~ 1% change). Also, for Pd 3d spectra, annealing induced only a minor difference (about 10% and 15% for 0.2 and 0.5 ML, respectively). Consequently, the Pd nanoparticles, anchored in HOPG defects, were thermally stable upon annealing to 300 °C.

The number of Pd atoms corresponding to 0.2 ML Pd/HOPG is 3.06 × 10^14^ atoms cm^−2^, that corresponding to 0.5 ML Pd/HOPG is 7.65 × 10^14^ atoms cm^−2^ (Table 1). The nominal Pd film thickness calculation is somewhat simplified, but it mainly serves for comparison with the current and previous [85] STM data. Even assuming 2D growth, the agreement with the STM data presented below is fairly good, however.

To directly determine the size and number density of the Pd nanoparticles after annealing to 300 °C, in situ STM images of the 0.2 (Fig. 3c) and 0.5 ML Pd/HOPG (Fig. 3g) model catalysts were taken at room temperature.

For the lower loading 0.2 ML Pd/HOPG, many Pd particles decorated steps on HOPG, but about 20% of the particles were located on flat terraces. The average number of Pd atoms per particle can then be calculated from the Pd amount deduced from XPS (3.06 × 10^14^ Pd atoms cm^−2^) and the Pd particle number density deduced from STM (7.94 × 10^11^ particles/cm^2^), as the latter is hardly affected by STM tip convolution effects [83, 84, 107], yielding 387 Pd atoms/particle. The average Pd particle size determined *directly* from STM was 4.3 nm (*c.f.* the size histogram in Fig. 3d) with a mean height of 0.5 nm. Although the apparent size may be larger than the actual one (due to tip convolution [83, 84, 107], the high aspect ratio of ~ 9 suggests a flat “raft-like” morphology (Fig. 1a), not unusual for metal islands/particles on HOPG [115–121] (note also the rather uniform STM contrast of the magnified particle). If one thus assumes a hemispherical shape with truncated top (hemispherical cap), a mean size of 4.3 nm would correspond to 292 Pd atoms/particle and 214 surface atoms/particle (note that these values are quite different from those obtained for the commonly used hemispherical shape [9, 74]). Considering the Pd particle number density from STM (7.94 × 10^11^ particles/cm^2^), the resulting total Pd amount is 2.32 × 10^14^ atoms cm^−2^. Note that this corresponds reasonably well to the Pd amount deduced from XPS (3.06 × 10^14^ Pd atoms cm^−2^). The number of Pd surface atoms in the sample (0.38 cm^2^) can then be calculated accordingly (Table 1).

An analogous analysis was carried out for the 0.5 ML Pd/HOPG model catalyst (Fig. 3g). More Pd particles were growing on the terraces and the densely packed larger Pd particles seem to cover nearly the entire support. Once more, based on the Pd amount from XPS (7.65 × 10^14^ Pd atoms cm^−2^) and the particle density (9.42 × 10^11^ cm^−2^) from STM (Table 1), every Pd particle should contain 812 atoms on average. The mean size (*c.f.* the size histogram in Fig. 3h) and height deduced from STM were 6.8 and 0.6 nm, respectively, indicating that every Pd particle contained on average 802 atoms and 518 surface atoms/particle. Considering the Pd particle number density from STM (9.42 × 10^11^ particles/cm^2^), the resulting total Pd amount is 7.56 × 10^14^ atoms cm^−2^ which again corresponds well to the Pd amount deduced from XPS (7.65 × 10^14^ Pd atoms cm^−2^). The number of Pd surface atoms in the sample (0.38 cm^2^) can once more be calculated accordingly (Table 1).

**Fig. 4:**
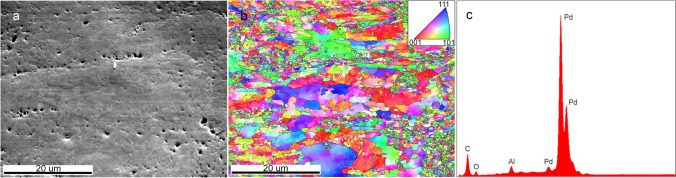
**a** SEM (scale bar is 20 μm), **b** EBSD (scale bar is 20 μm) with crystallographic color code and **c** EDX analysis of polycrystalline Pd foil

As for both types of analysis quite similar average numbers of Pd atoms/particle were obtained, the particle characteristics directly determined by STM will be used for further calculations below. Kettner et al. [83] also reported that STM overestimated the nanoparticle volume by only 8%. Altogether, this reassured us to use the direct STM characterization as input for TOF calculations. Nevertheless, the turnover frequencies (TOFs) described below should be considered an upper limit, especially for particles with smaller size/height ratio. For Pd foil, SEM and EBSD revealed a polycrystalline surface, with EDX confirming the absence of impurities (Fig. 4; Al signal due to the sample holder). For XPS spectra of Pd (and Pt) foil refer to [108].

All structural data of the model catalysts are summarized in Table 1. In agreement with previous nucleation studies [122], the particle number density is roughly 5 times smaller for Pd than for Pt, due the higher Pd mobility during growth. For the current metal exposures, the Pd nanoparticles had about twice the size of Pt nanoparticles.

### Flow Microreactor Studies of C_2_H_4_ Hydrogenation on Pd- and Pt-Based Catalysts

After characterization by XPS/STM and/or SEM/EBSD/EDX, every Pd-based model catalyst was transferred in air to the UHV-compatible microreactor, which enables to determine catalytic properties under atmospheric pressure flow conditions [85, 108]. As mentioned, the model catalyst was mounted inside the stainless steel microreactor cell with a thermocouple attached. After degassing and cleaning in UHV (annealing in low pressures of O_2_, H_2_), the catalyst was moved to the lower level of the UHV chamber, where the microreactor assembly was located (the reaction cell then locks into the oven front piece). The microreactor cell was subsequently closed by the gas supply tube. In the sealed microreactor, the catalysts were additionally cleaned by oxidative and reductive treatments (at 250 °C, at a flow rate of 0.2 ml/min O_2_ or 15 ml/min H_2_, both with 5 ml/min Ar;5 and 60 min, respectively), to remove any potential carbon species from Pd (which may originate from air transfer or ion-bombardment [81–83]). As discussed in Sect. 2.1, such treatments were demonstrated not to change the mean nanoparticle size or distribution.

Ethylene hydrogenation to ethane is a classical reaction following Langmuir–Hinshelwood kinetics via stepwise hydrogenation, a mechanism proposed by Horiuti and Polanyi in 1934 [123]. Although the reaction does not require selectivity (if one neglects C_2_H_4_ decomposition), it is a valuable test for benchmarking different catalysts. Along these lines, C_2_H_4_ adsorption, C_2_H_4_/H coadsorption and the effects of subsurface hydrogen and Pd-hydride formation were extensively studied [2–4, 29, 30, 33, 47, 62, 67, 74, 124, 125].

**Fig. 5:**
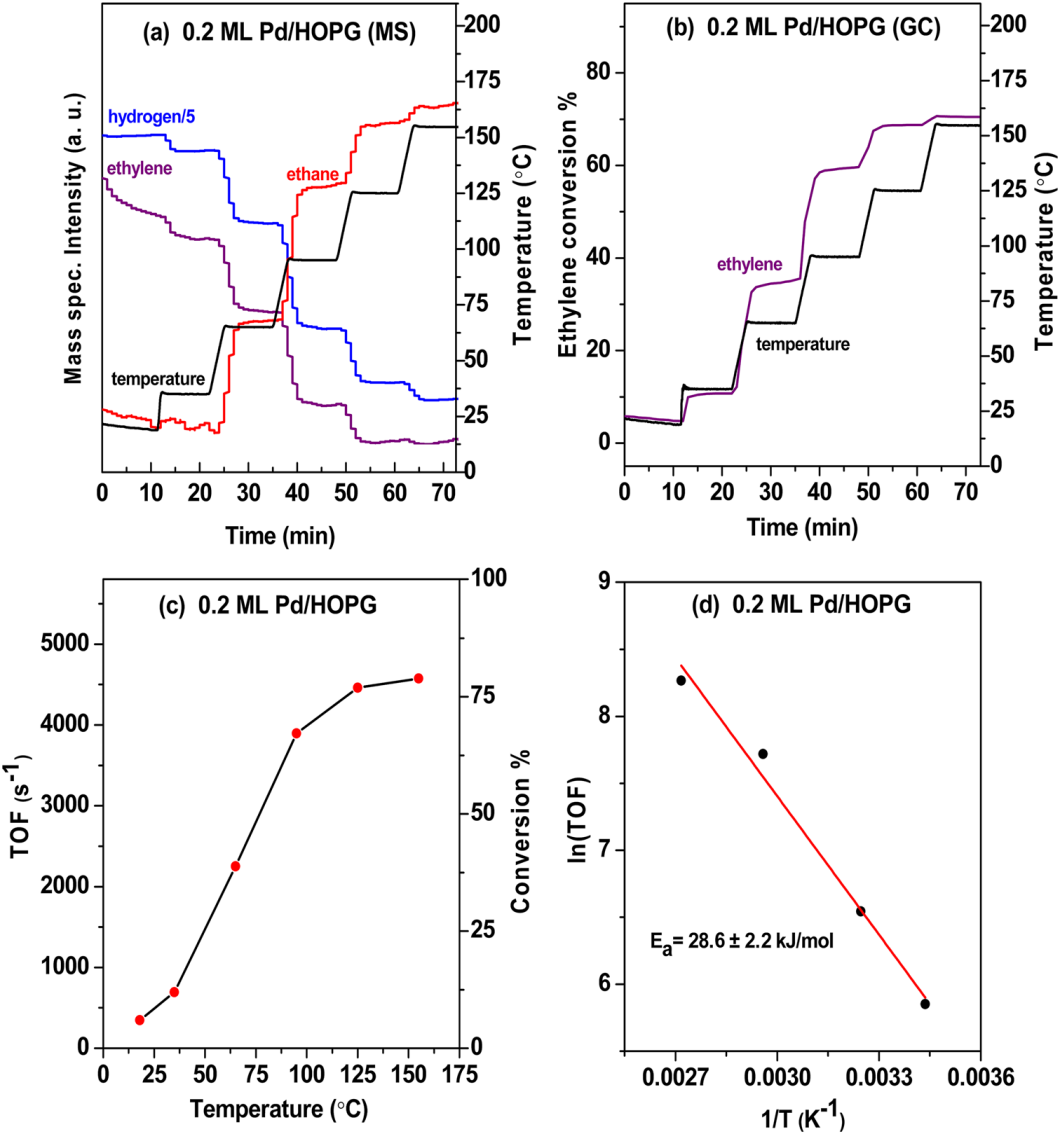
Flow microreactor study of ethylene hydrogenation on 0.2 ML Pd/HOPG model catalyst (4.3 nm Pd particles) at atmospheric pressure. **a**, **b** The gas composition at the reactor outlet was analyzed by a differentially-pumped mass spectrometer (MS) and quantified by a gas chromatograph (GC). **c** TOFs vs. temperature. **d** Arrhenius plot

“Blank” reaction studies with HOPG alone indicated zero activity of the support, agreeing well with our previous powder study of pure graphene nanoplatelets (GNPs) or activated carbon [47]. Figure 5 displays microreactor results of C_2_H_4_ hydrogenation on the 0.2 ML Pd/HOPG model catalyst (4.3 nm Pd particles) with a flow rate of 1 ml/min C_2_H_4_, 1 ml/min H_2_, and 12 ml/min Ar. The catalyst was heated stepwise from room temperature to 155 °C, with a temperature ramp of 10 °C/min and isothermal periods of 10 min. The gas composition at the reactor outlet was analyzed by MS (Fig. 5a) and quantified by GC (Fig. 5b).

According to the GC measurements at different temperatures, the ethylene conversions were ∼11% and ∼60% at 35 and 95 °C, respectively. The temperature-dependent turnover frequencies (TOFs, the number of C_2_H_4_ molecules converted per second and per Pd surface atom), with the total number of Pd surface atoms calculated from STM, are presented in Fig. 5c. The activation energy for ethylene hydrogenation on 0.2 ML Pd/HOPG was 28.6 ± 2.2 kJ/mol, deduced from the Arrhenius plot in Fig. 5d (Table 1).

**Fig. 6:**
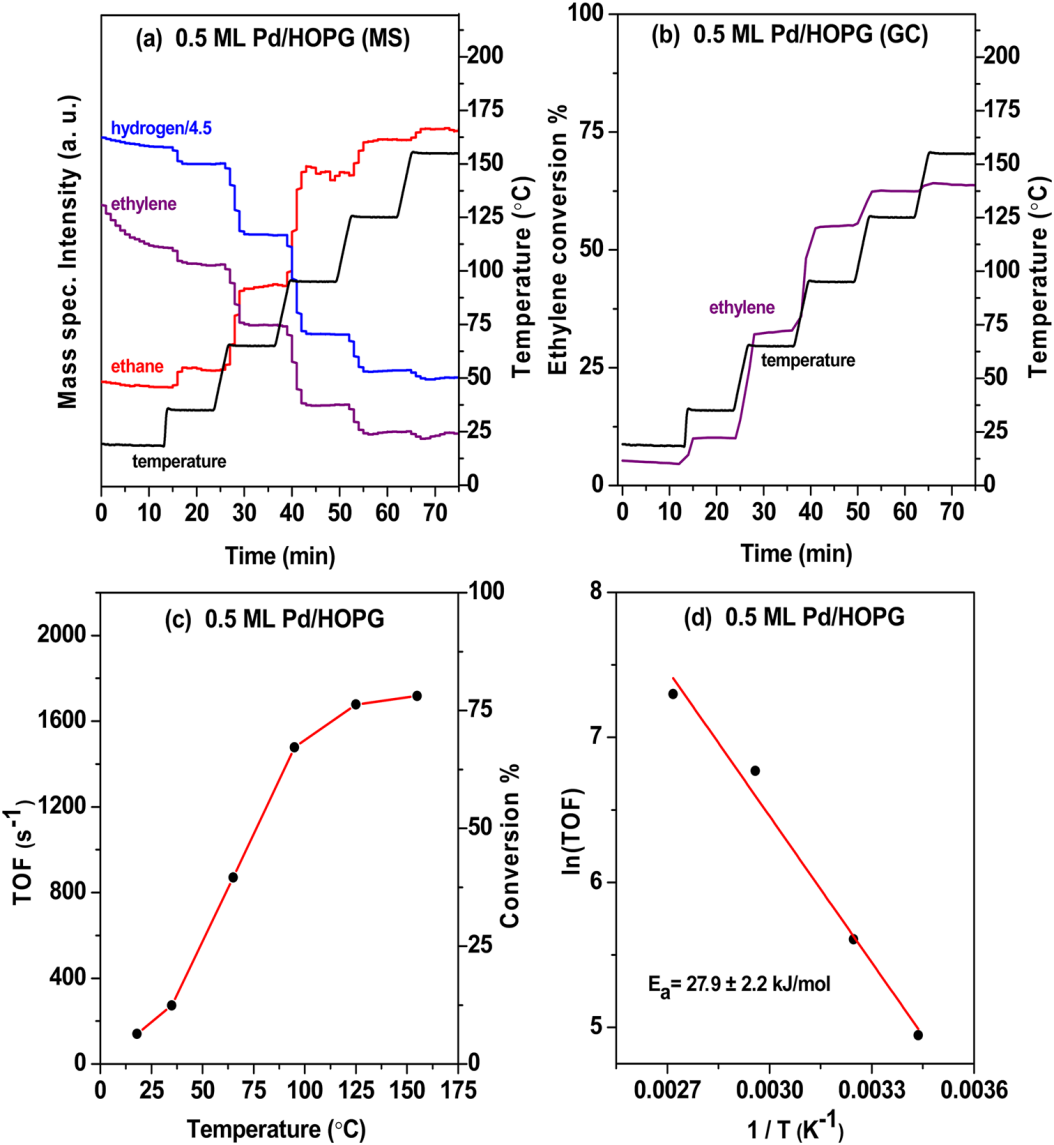
Flow microreactor study of ethylene hydrogenation on 0.5 ML Pd/HOPG model catalyst (6.8 nm Pd particles) at atmospheric pressure. **a**, **b** The gas composition at the reactor outlet was analyzed by a differentially-pumped mass spectrometer (MS) and quantified by a gas chromatograph (GC). **c** TOFs vs. temperature. **d** Arrhenius plot

Figure 6 displays results from the corresponding ethylene hydrogenation on the 0.5 ML Pd/HOPG model catalyst (6.8 nm Pd particles), again with a flow rate of 1 ml/min C_2_H_4_, 1 ml/min H_2_, and 12 ml/min Ar (MS in Fig. 6a and GC in Fig. 6b). Based on the GC measurements at different temperatures, the ethylene conversions were ∼12% and ∼65% at 35 and 95 °C, respectively. The temperature-dependent TOFs are shown in Fig. 6c. The activation energy of 27.9 ± 2.2 kJ/mol for ethylene hydrogenation on 0.5 ML Pd/HOPG model catalyst was obtained from the Arrhenius plot in Fig. 6d (Table 1).

**Fig. 7:**
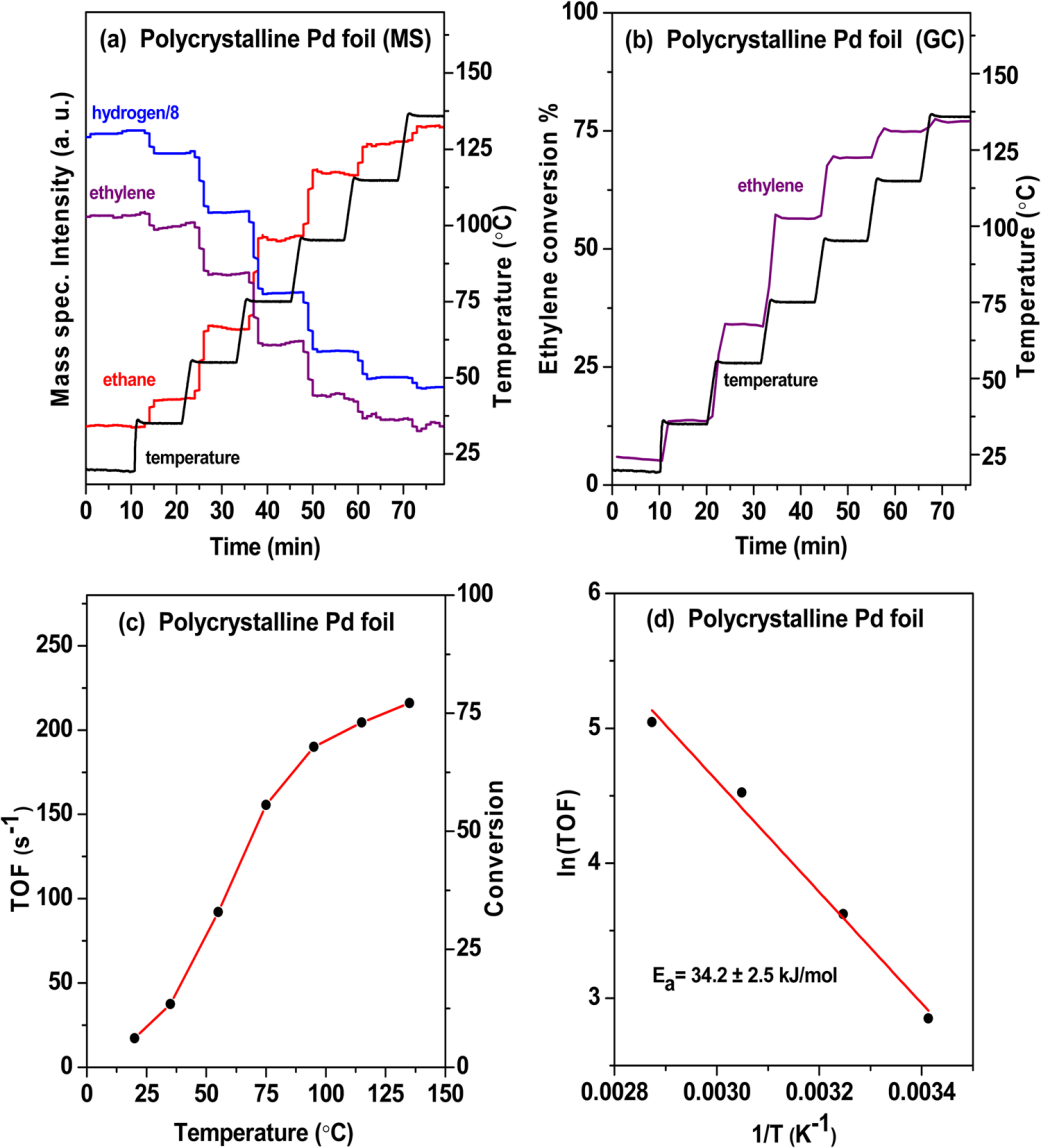
Flow microreactor study of ethylene hydrogenation on Pd foil at atmospheric pressure. **a**, **b** The gas composition at the reactor outlet was analyzed by differentially-pumped MS and quantified by GC. **c** TOFs vs. temperature. **d** Arrhenius plot

To discriminate the role of the nano-sized Pd particles and of the support, polycrystalline (unsupported) Pd foil was examined under the same conditions as well (Fig. 7). Reactants and products were once more analyzed by MS (Fig. 7a) and quantified by GC (Fig. 7b). Based on the GC measurements at different temperatures, the ethylene conversions were ∼13% and ∼66% at 35 and 95 °C, respectively. The temperature-dependent TOFs are shown in Fig. 7c. The Arrhenius plot (Fig. 7d) yielded an activation energy of 34.2 ± 2.5 kJ/mol, which is significantly higher than that of HOPG supported Pd nanoparticles.

For all model catalysts, the performance was reversible and stable up to ~150° C. Above that, catalyst deactivation occurred with time (likely by carbon poisoning) [108].

Although ethylene hydrogenation is considered a structure-insensitive reaction, the TOFs of Pd/HOPG at ~ 50–100 °C show a clear trend, with the nanoparticles being about 20- to 8-times more active than the Pd foil, which is astonishing. The activation energy found for Pd particles on HOPG (~ 27 ± 2 kJ/mol) is in good agreement with that reported for supported Pd particles (~ 28–32 kJ/mol) [47], but it is significantly lower than that on bulk-Pd foil (~ 34 ± 2 kJ/mol). It should also be noted that just the front side of the metal foil was considered to be active as the unsputtered/not-annealed backside was clamped to the reactor wall. However, if the backside would contribute some activity, the TOFs of the foil would be even smaller.

Several reasons may account for the difference between nanoparticles and foil. Clearly, the Pd nanoparticles exhibit even more low coordinated metal sites than polycrystalline Pd foil does. Nevertheless, in a previous study similarly shaped Pd nanoparticles were supported either by carbon (GNPs) or alumina, but the GNP-supported Pd nanoparticles still showed much higher activity above 40 °C [47]. This rather rules out a surface structure effect such as that recently described for smooth and stepped Pd facets [8, 9]. Furthermore, hydrogen dissolution in Pd, i.e., the formation of Pd hydrides, should also not depend on the type of support.

**Fig. 8:**
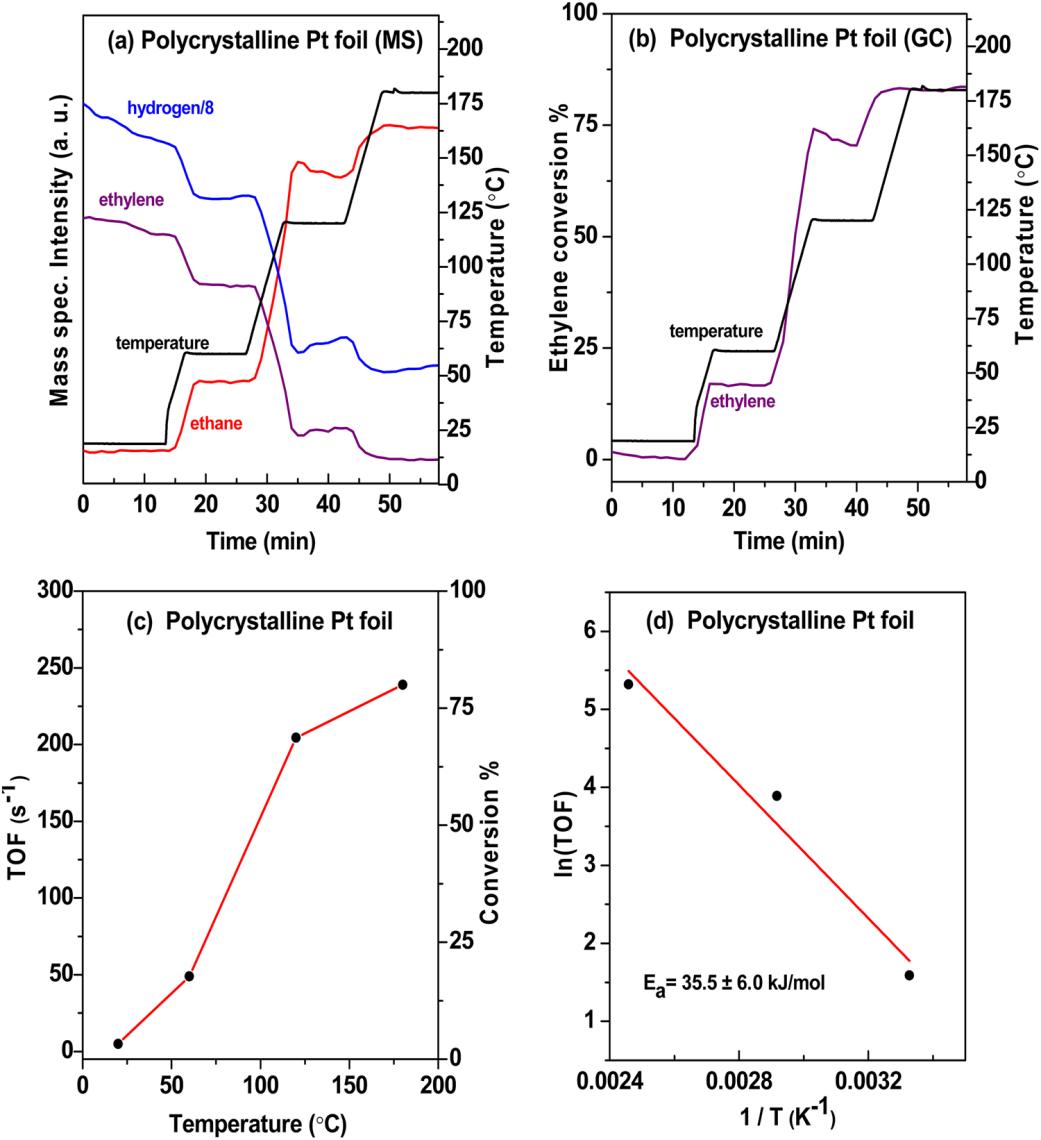
Flow microreactor study of ethylene hydrogenation on Pt foil at atmospheric pressure. **a**, **b** The gas composition at the reactor outlet was analyzed by differentially-pumped MS and quantified by GC. **c** TOFs and conversion vs. temperature. **d** Arrhenius plot

To fully exclude an effect of metal hydrides, ethylene hydrogenation was also carried out on a polycrystalline Pt foil and contrasted to our previous results on HOPG-supported Pt nanoparticles (XPS and STM results were described in [85]), as for Pt one does not expect a pronounced influence of dissolved hydrogen. Figure 8 shows the measured reactivity profile of Pt foil (1 ml/min C_2_H_4_, 4 ml/min H_2_, and 12 ml/min Ar, heated stepwise from 25 to 250 °C with a temperature ramp of 10 °C/min and isothermal periods of 10 min). The gas composition at the reactor outlet was analogously analyzed by MS (Fig. 8a) and quantified by GC (Fig. 8b).

The GC measurements indicated ethylene conversions of ∼17% and ∼71% at 60 and 120 °C, respectively (for C_2_H_4_/H_2_ of 1 they were about 50 % less). The temperature-dependent TOFs are shown in Fig. 8c. The activation energy of ethylene hydrogenation on polycrystalline Pt foil, obtained by an Arrhenius plot, was 35.5 ± 6.0 kJ/mol (Fig. 8d). Similar as for Pd, for bulk-Pt foil the TOFs at a given temperature were lower and the E_a_ was about 10 kJ/mol higher than for HOPG-supported Pt nanoparticles (previous results for 0.31 and 0.74 ML Pt/HOPG [85], adapted in light of the current GC analysis, are included in Table 1).

Based on the arguments presented, an effect of the nanoparticle shape/surface structure (smooth vs. rough facets [8, 9]) or of metal hydrides can be ruled out. Apparently, both for Pd and Pt, there is a pronounced effect of the HOPG support on C_2_H_4_ hydrogenation: the nanoparticles were more active than polycrystalline foil. It is thus suggested that additional hydrogen can be accommodated at the metal/carbon interface, resulting in higher activity [47]. In other words, the interfacial hydrogen may facilitate hydrogen availability at the metal/carbon phase boundary. The raft-like morphology of the Pd and Pt deposits on the HOPG support, leading to size/height aspect ratios up to ~ 10 (for perimeter atoms/surface atoms see Table 1), is certainly beneficial, but the exact origin of this effect has been debated.

Hydrogen adsorption/absorption/desorption has been repeatedly studied for supported Pt and Pd catalysts by temperature programmed methods, NMR and inelastic neutron scattering (INS) [2, 3, 33, 62, 64, 65, 124–128]. In ^1^H NMR studies of hydrogen chemisorption over Pt/SiO_2_ a specific resonance was attributed to hydrogen at the Pt-silica interface [64, 127]. Using INS, it was demonstrated that the carbon support can critically affect the rate of release of hydrogen stored in Pd particles [65, 126].

The strongest support for this phenomenon comes from a combined experimental and theoretical study of carbon-supported Pd hydrogenation catalysts (~ 5 nm Pd particles) by Rao et al. [63], who suggested the charge transfer between carbon support and the active metal to be responsible for fine tuning of the electronic structure of the catalytic centers, hence affecting their catalytic performance. DFT modeling revealed that this direct electronic effect is restricted to the proximity of the interface, because it dissipates beyond two metal layers. The binding energy of adsorbates such as H, C, O, OH, and CH can still be influenced up to 5 metal layers above the support, which has been attributed to changes of the d-band of the metal [63]. Another DFT study of support effects in C_2_H_2_ semi-hydrogenation reported deviations of ~ 20 kJ/mol for ethylene adsorption energies for a two-layer Pd cluster, strongly changing catalytic performance [129]. The specific role of metal/support perimeter sites was also demonstrated for other carbon and oxide supports [130–136]. Altogether, the previous and the current model studies indicate the importance of the nature and structure of the metal/carbon interface in affecting hydrogen and hydrocarbon supply for subsequent reactions, an aspect that should receive further attention in the future.

## Conclusions

Pd/HOPG model catalysts, with Pd nanoparticles of raft-like morphology and different mean size, were grown in UHV and annealed to 300 °C (to guarantee thermal stability under reaction conditions up to 155 °C). The defect density of ion-bombarded HOPG and the nominal thickness of the Pd overlayer were determined in situ by XPS. STM directly provided the Pd particle number density and mean particle size/height, with moderate tip convolution effects. Flow microreactor studies of ethylene hydrogenation to ethane at atmospheric pressure, comparing two different Pd/HOPG model catalysts (4.3 and 6.8 nm mean Pd particle size) with polycrystalline Pd foil, revealed a pronounced beneficial effect of the HOPG support.

Interestingly, the nano-sized and HOPG-supported Pd particles exhibited higher specific activity (TOF) and lower activation energy (E_a_) than bulk-Pd foil, despite C_2_H_4_ hydrogenation being considered structure-insensitive. In a previous study on powder catalysts [47], Pd nanoparticles were more active when supported on carbon, than when supported on oxide. Altogether, this suggests a support effect, rather than an influence of the metal atom coordination (particle shape/roughess). Similarly, if formed, Pd hydrides should be the same, independent of the support.

Analogous C_2_H_4_ hydrogenation studies for bulk-Pt foil and Pt/HOPG also revealed a higher activity and lower E_a_’s for carbon-supported Pt nanoparticles. As Pt is unable to significantly dissolve hydrogen, this once more points to a carbon support effect.

Accordingly, the increased activity and lower activation energy of HOPG-supported metal nanoparticles is assigned to the metal/carbon interface. Charge transfer between the support and the active metal may modify the electronic structure of the catalytic centers, thus affecting the binding energies of adsorbates. Further kinetic, operando spectroscopic [101] and theoretical [8, 9] studies are required to better characterize the relevant interfacial hydrogen and hydrocarbon species, which will be very challenging, though.

## Data Availability

The datasets generated during and/or analysed during the current study are available from the corresponding author on reasonable request.

## References

[1] Beebe TP, Yates JT (1986). An in situ infrared spectroscopic investigation of the role of ethylidyne in the ethylene hydrogenation reaction on palladium/alumina. J Am Chem Soc.

[2] Doyle AM, Shaikhutdinov SK, Jackson SD, Freund H-J (2003). Hydrogenation on metal surfaces: why are nanoparticles more active than single crystals?. Angew Chem Int Ed.

[3] Morkel M, Rupprechter G, Freund H-J (2005). Finite size effects on supported Pd nanoparticles: interaction of hydrogen with CO and C_2_H_4_. Surf Sci.

[4] Freund H-J, Bäumer M, Libuda J, Risse T, Rupprechter G, Shaikhutdinov S (2003). Preparation and characterization of model catalysts: from ultrahigh vacuum to in situ conditions at the atomic dimension. J Catal.

[5] Zaera F, Somorjai GA (1984). Hydrogenation of ethylene over platinum (111) single-crystal surfaces. J Am Chem Soc.

[6] Stacchiola D, Azad S, Burkholder L, Tysoe WT (2001). An investigation of the reaction pathway for ethylene hydrogenation on Pd(111). J Phys Chem B.

[7] Molero H, Stacchiola D, Tysoe WT (2005). The kinetics of ethylene hydrogenation catalyzed by metallic palladium. Catal Lett.

[8] Markova VK, Philbin JP, Zhao W, Genest A, Silvestre-Albero J, Rupprechter G, Rösch N (2018). Catalytic transformations of 1-butene over palladium. A combined experimental and theoretical study. ACS Catal.

[9] Genest A, Silvestre-Albero J, Li W-Q, Rösch N, Rupprechter G (2021). The origin of the particle-size-dependent selectivity in 1-butene isomerization and hydrogenation on Pd/Al_2_O_3_ catalysts. Nat Commun.

[10] Brandt B, Fischer J-H, Ludwig W, Libuda J, Zaera F, Schauermann S, Freund H-J (2008). Isomerization and hydrogenation of cis-2-butene on Pd model catalyst. J Phys Chem C.

[11] Silvestre-Albero J, Rupprechter G, Freund H-J (2005). Atmospheric pressure studies of selective 1,3-butadiene hydrogenation on Pd single crystals: effect of CO addition. J Catal.

[12] Bertolini JC, Delichere P, Khanra BC, Massardier J, Noupa C, Tardy B (1990). Electronic properties of supported Pd aggregates in relation with their reactivity for 1,3-butadiene hydrogenation. Catal Lett.

[13] Silvestre-Albero J, Rupprechter G, Freund H-J (2006). From Pd nanoparticles to single crystals: 1,3-butadiene hydrogenation on well-defined model catalysts. Chem Commun.

[14] Tardy B (1991). Catalytic hydrogenation of 1,3-butadiene on Pd particles evaporated on carbonaceous supports: particle size effect. J Catal.

[15] Constant L, Ruiz P, Abel M, Robach Y, Porte L, Bertolini JC (2000). Pd deposited on Cu(110): a highly performant catalyst for the 1,3-butadiene hydrogenation reaction. Top Catal.

[16] Yan H, Cheng H, Yi H, Lin Y, Yao T, Wang C, Li J, Wei S, Lu J (2015). Single-Atom Pd_1_/graphene catalyst achieved by atomic layer deposition: remarkable performance in selective hydrogenation of 1,3-butadiene. J Am Chem Soc.

[17] Sarkany A, Zsoldos Z, Furlong B, Hightower JW, Guczi L (1993). Hydrogenation of 1-butene and 1,3-butadiene mixtures over Pd/ZnO catalysts. J Catal.

[18] Silvestre-Albero J, Rupprechter G, Freund H-J (2006). Atmospheric pressure studies of selective 1,3-butadiene hydrogenation on well-defined Pd/Al_2_O_3_/NiAl(110) model catalysts: effect of Pd particle size. J Catal.

[19] Bos ANR, Botsma ES, Foeth F, Sleyster HWJ, Westerterp KR (1993). A kinetic study of the hydrogenation of ethyne and ethene on a commercial Pd/Al_2_O_3_ catalyst. Chem Eng Process.

[20] Khan NA, Shaikhutdinov S, Freund H-J (2006). Acetylene and ethylene hydrogenation on alumina supported Pd-Ag model catalysts. Catal Lett.

[21] Zea H, Lester K, Datye AK, Rightor E, Gulotty R, Waterman W, Smith M (2005). The influence of Pd–Ag catalyst restructuring on the activation energy for ethylene hydrogenation in ethylene–acetylene mixtures. Appl Catal A.

[22] Lear T, Marshall R, Gibson EK, Schutt T, Klapötke TM, Rupprechter G, Freund H-J, Winfield JM, Lennon D (2005). A model high surface area alumina-supported palladium catalyst. Phys Chem Chem Phys.

[23] Kennedy DR, Webb G, Jackson SD, Lennon D (2004). Propyne hydrogenation over alumina-supported palladium and platinum catalysts. Appl Catal A.

[24] McInroy AR, Uhl A, Lear T, Klapötke TM, Shaikhutdinov S, Schauermann S, Rupprechter G, Freund H-J, Lennon D (2011). Morphological and chemical influences on alumina-supported palladium catalysts active for the gas phase hydrogenation of crotonaldehyde. J Chem Phys.

[25] Dostert K-H, O’Brien CP, Mirabella F, Ivars-Barceló F, Attia S, Spadafora E, Schauermann S, Freund H-J (2017). Selective partial hydrogenation of acrolein on Pd: a mechanistic study. ACS Catal.

[26] Arnold H, Döbert F, Gaube J, Selective hydrogenation of hydrocarbons. In: Handbook of heterogeneous catalysis, pp 3266–3284

[27] Bertolini JC, Rousset JL, Bréchignac C, Houdy P, Lahmani M (2007). Reactivity of metal nanoparticles. Nanomaterials and nanochemistry.

[28] Lan X, Wang T (2020). Highly selective catalysts for the hydrogenation of unsaturated aldehydes: a review. ACS Catal.

[29] Zaera F (2017). The surface chemistry of metal-based hydrogenation catalysis. ACS Catal.

[30] Aleksandrov HA, Vines F, Ludwig W, Schauermann S, Neyman KM (2013). Tuning the surface chemistry of Pd by atomic C and H: a microscopic picture. Chemistry.

[31] Papp C (2016). From flat surfaces to nanoparticles: In situ studies of the reactivity of model catalysts. Catal Lett.

[32] Freund H-J, Nilius N, Risse T, Schauermann S (2014). A fresh look at an old nano-technology: catalysis. Phys Chem Chem Phys.

[33] Schauermann S, Freund H-J (2015). model approach in heterogeneous catalysis: Kinetics and thermodynamics of surface reactions. Acc Chem Res.

[34] Schauermann S, Nilius N, Shaikhutdinov S, Freund H-J (2013). Nanoparticles for heterogeneous catalysis: new mechanistic insights. Acc Chem Res.

[35] Rupprechter G, Somorjai GA (1997). Palladium-catalyzed hydrogenation without hydrogen - the hydrodechlorination of chlorofluorocarbons with solid-state hydrogen over the palladium(111) crystal-surface and its implications. Catal Lett.

[36] Auer E, Freund A, Pietsch J, Tacke T (1998). Carbons as supports for industrial precious metal catalysts. Appl Catal A.

[37] Benavidez AD, Burton PD, Nogales JL, Jenkins AR, Ivanov SA, Miller JT, Karim AM, Datye AK (2014). Improved selectivity of carbon-supported palladium catalysts for the hydrogenation of acetylene in excess ethylene. Appl Catal A.

[38] McMillan L, Gilpin LF, Baker J, Brennan C, Hall A, Lundie DT, Lennon D (2016). The application of a supported palladium catalyst for the hydrogenation of aromatic nitriles. J Mol Catal A.

[39] McAllister MI, Boulho C, McMillan L, Gilpin LF, Wiedbrauk S, Brennan C, Lennon D (2018). The production of tyramine via the selective hydrogenation of 4-hydroxybenzyl cyanide over a carbon-supported palladium catalyst. RSC Adv.

[40] Wei Z, Pan R, Hou Y, Yang Y, Liu Y (2015). Graphene-supported Pd catalyst for highly selective hydrogenation of resorcinol to 1, 3-cyclohexanedione through giant pi-conjugate interactions. Sci Rep.

[41] Bugaev AL, Usoltsev OA, Lazzarini A, Lomachenko KA, Guda AA, Pellegrini R, Carosso M, Vitillo JG, Groppo E, van Bokhoven JA, Soldatov AV, Lamberti C (2018). Time-resolved operando studies of carbon supported Pd nanoparticles under hydrogenation reactions by X-ray diffraction and absorption. Faraday Discuss.

[42] Rana S, Maddila S, Jonnalagadda SB (2015). Synthesis and characterization of Pd(II) dispersed over diamine functionalized graphene oxide and its scope as a catalyst for selective oxidation. Catal Sci Technol.

[43] Maiyalagan T, Wang X, Manthiram A (2014). Highly active Pd and Pd–Au nanoparticles supported on functionalized graphene nanoplatelets for enhanced formic acid oxidation. RSC Adv.

[44] Rana S, Maddila S, Yalagala K, Jonnalagadda SB (2015). Organo functionalized graphene with Pd nanoparticles and its excellent catalytic activity for Suzuki coupling reaction. Appl Catal A.

[45] Lee H, Paeng K, Kim IS (2018). A review of doping modulation in graphene. Synth Met.

[46] Deerattrakul V, Yigit N, Rupprechter G, Kongkachuichay P (2019). The roles of nitrogen species on graphene aerogel supported Cu-Zn as efficient catalysts for CO_2_ hydrogenation to methanol. Appl Catal A.

[47] Dobrezberger K, Bosters J, Moser N, Yigit N, Nagl A, Föttinger K, Lennon D, Rupprechter G (2020). Hydrogenation on palladium nanoparticles supported by graphene nanoplatelets. J Phys Chem C.

[48] Yao Y, Fu Q, Zhang YY, Weng X, Li H, Chen M, Jin L, Dong A, Mu R, Jiang P, Liu L, Bluhm H, Liu Z, Zhang SB, Bao X (2014). Graphene cover-promoted metal-catalyzed reactions. Proc Nat Acad Sci USA.

[49] Xu C, Wang X, Zhu J (2008). Graphene−metal particle nanocomposites. J Phys Chem C.

[50] Baghayeri M, Veisi H, Veisi H, Maleki B, Karimi-Maleh H, Beitollahi H (2014). Multi-walled carbon nanotubes decorated with palladium nanoparticles as a novel platform for electrocatalytic sensing applications. RSC Adv.

[51] Li J, Zhu QL, Xu Q (2015). Pd nanoparticles supported on hierarchically porous carbons derived from assembled nanoparticles of a zeolitic imidazolate framework (ZIF-8) for methanol electrooxidation. Chem Commun.

[52] Zhao S, Mu R, Ning Y, Fu Q, Bao X (2020). Modulating electronic structure of graphene overlayers through electrochemical intercalation. Appl Surf Sci.

[53] Hsieh SH, Hsu MC, Liu WL, Chen WJ (2013). Study of Pt catalyst on graphene and its application to fuel cell. Appl Surf Sci.

[54] Marinkas A, Arena F, Mitzel J, Prinz GM, Heinzel A, Peinecke V, Natter H (2013). Graphene as catalyst support: the influences of carbon additives and catalyst preparation methods on the performance of PEM fuel cells. Carbon.

[55] Bowker M, Morgan C, Perkins N, Holroyd R, Fourre E, Grillo F, MacDowall A (2005). Ethene adsorption, dehydrogenation and reaction with Pd(110): Pd as a carbon 'sponge'. J Phys Chem B.

[56] Gabasch H, Hayek K, Klötzer B, Knop-Gericke A, Schlögl R (2006). Carbon incorporation in Pd(111) by adsorption and dehydrogenation of ethene. J Phys Chem B.

[57] Teschner D, Borsodi J, Wootsch A, Révay Z, Hävecker M, Knop-Gericke A, Jackson SD, Schlögl R (2008). The roles of subsurface carbon and hydrogen in palladium-catalyzed alkyne hydrogenation. Science.

[58] Teschner D, Pestryakov A, Kleimenov E, Hävecker M, Bluhm H, Sauer H, Knop-Gericke A, Schlögl R (2005). High-pressure X-ray photoelectron spectroscopy of palladium model hydrogenation catalysts. Part 2: hydrogenation of trans-2-pentene on palladium. J Catal.

[59] Borasio M, Rodriguez de la Fuente O, Rupprechter G, Freund H-J (2005). In situ studies of methanol decomposition and oxidation on Pd(111) by PM-IRAS and XPS spectroscopy. J Phys Chem B.

[60] Morkel M, Kaichev VV, Rupprechter G, Freund H-J, Prosvirin IP, Bukhtiyarov VI (2004). Methanol dehydrogenation and formation of carbonaceous overlayers on Pd(111) studied by high-pressure SFG and XPS spectroscopy. J Phys Chem B.

[61] McNamara JM, Jackson SD, Lennon D (2003). Butane dehydrogenation over Pt/alumina: activation, deactivation and the generation of selectivity. Catal Today.

[62] Wilde M, Fukutani K, Ludwig W, Brandt B, Fischer JH, Schauermann S, Freund H-J (2008). Influence of carbon deposition on the hydrogen distribution in Pd nanoparticles and their reactivity in olefin hydrogenation. Angew Chem Int Ed.

[63] Rao RG, Blume R, Hansen TW, Fuentes E, Dreyer K, Moldovan S, Ersen O, Hibbitts DD, Chabal YJ, Schlögl R, Tessonnier JP (2017). Interfacial charge distributions in carbon-supported palladium catalysts. Nat Commun.

[64] Chesters MA, Packer KJ, Viner HE, Wright MAP, Lennon D (1995). ^1^H NMR of hydrogen chemisorbed on silica-supported platinum particles: an evaluation of different models. J Chem Soc Faraday Trans.

[65] Möbus K, Grünewald E, Wieland SD, Parker SF, Albers PW (2014). Palladium-catalyzed selective hydrogenation of nitroarenes: Influence of platinum and iron on activity, particle morphology and formation of β-palladium hydride. J Catal.

[66] Neyman KM, Schauermann S (2010). Hydrogen diffusion into palladium nanoparticles: pivotal promotion by carbon. Angew Chem Int Ed.

[67] Aleksandrov HA, Kozlov SM, Schauermann S, Vayssilov GN, Neyman KM (2014). How absorbed hydrogen affects the catalytic activity of transition metals. Angew Chem Int Ed.

[68] Freund H-J (1997). Adsorption of gases on complex solid surfaces. Angew Chem Int Ed.

[69] Pacchioni G, Freund H-J (2018). Controlling the charge state of supported nanoparticles in catalysis: Lessons from model systems. Chem Soc Rev.

[70] Goodman DW (1995). Model studies in catalysis using surface science probes. Chem Rev.

[71] Campbell CT (1997). Ultrathin metal films and particles on oxide surfaces: structural, electronic and chemisorptive properties. Sci Rep.

[72] Henry CR (1998). Surface studies of supported model catalysts. Surf Sci Rep.

[73] Pan Q, Li L, Shaikhutdinov S, Fujimori Y, Hollerer M, Sterrer M, Freund H-J (2018). Model systems in heterogeneous catalysis: towards the design and understanding of structure and electronic properties. Faraday Discuss.

[74] Rupprechter G (2007). Sum frequency generation and polarization–modulation infrared reflection absorption spectroscopy of functioning model catalysts from ultrahigh vacuum to ambient pressure. Adv Catal.

[75] Dellwig T, Rupprechter G, Unterhalt H, Freund H-J (2000). Bridging the pressure and materials gaps: high pressure sum frequency generation study on supported Pd nanoparticles. Phys Rev Lett.

[76] Wang D (2003). Silicide formation on a Pt/SiO_2_ model catalyst studied by TEM, EELS, and EDXS. J Catal.

[77] Borchert H, Jürgens B, Zielasek V, Rupprechter G, Giorgio S, Henry CR, Bäumer M (2007). Pd nanoparticles with highly defined structure on MgO as model catalysts: An FTIR study of the interaction with CO, O_2_, and H_2_ under ambient conditions. J Catal.

[78] Somorjai GA, Rupprechter G (1998). The flexible surface: molecular studies explain the extraordinary diversity of surface chemical properties. J Chem Educ.

[79] Demidov DV, Prosvirin IP, Sorokin AM, Bukhtiyarov VI (2011). Model Ag/HOPG catalysts: preparation and STM/XPS study. Catal Sci Technol.

[80] Favaro M, Agnoli S, Perini L, Durante C, Gennaro A, Granozzi G (2013). Palladium nanoparticles supported on nitrogen-doped HOPG: a surface science and electrochemical study. Phys Chem Chem Phys.

[81] Bukhtiyarov AV, Prosvirin IP, Bukhtiyarov VI (2016). XPS/STM study of model bimetallic Pd-Au/HOPG catalysts. Appl Surf Sci.

[82] Bukhtiyarov AV, Prosvirin IP, Saraev AA, Klyushin AYu, Knop-Gericke A, Bukhtiyarov VI (2018). In situ formation of the active sites in Pd–Au bimetallic nanocatalysts for CO oxidation: NAP (near ambient pressure) XPS and MS study. Faraday Discuss.

[83] Kettner M, Stumm C, Schwarz M, Schuschke C, Libuda J (2019). Pd model catalysts on clean and modified HOPG: growth, adsorption properties, and stability. Surf Sci.

[84] Hohner C, Kettner M, Stumm C, Blaumeiser D, Wittkämper H, Grabau M, Schwarz M, Schuschke C, Lykhach Y, Papp C, Steinrück H-P, Libuda J (2020). Pt–Ga model SCALMS on modified HOPG: thermal behavior and stability in UHV and under near-ambient conditions. J Phys Chem C.

[85] Motin AM, Haunold T, Bukhtiyarov AV, Bera A, Rameshan C, Rupprechter G (2018). Surface science approach to Pt/carbon model catalysts: XPS, STM and microreactor studies. Appl Surf Sci.

[86] N'Diaye AT, Bleikamp S, Feibelman PJ, Michely T (2006). Two-dimensional Ir cluster lattice on a graphene moire on Ir(111). Phys Rev Lett.

[87] Franz D, Runte S, Busse C, Schumacher S, Gerber T, Michely T, Mantilla M, Kilic V, Zegenhagen J, Stierle A (2013). Atomic structure and crystalline order of graphene-supported Ir nanoparticle lattices. Phys Rev Lett.

[88] Wang B, Yoon B, Konig M, Fukamori Y, Esch F, Heiz U, Landman U (2012). Size-selected monodisperse nanoclusters on supported graphene: bonding, isomerism, and mobility. Nano Lett.

[89] Gotterbarm K, Steiner C, Bronnbauer C, Bauer U, Steinrück H-P, Maier S, Papp C (2014). Graphene-templated growth of Pd nanoclusters. J Phys Chem C.

[90] Gerber T, Knudsen J, Feibelman PJ, Granas E, Stratmann P, Schulte K, Andersen JN, Michely T (2013). CO-induced Smoluchowski ripening of Pt cluster arrays on the graphene/Ir(111) moire. ACS Nano.

[91] Linas S, Jean F, Zhou T, Albin C, Renaud G, Bardotti L, Tournus F (2015). Moiré induced organization of size-selected Pt clusters soft landed on epitaxial graphene. Sci Rep.

[92] Gotterbarm K, Spath F, Bauer U, Bronnbauer C, Steinrück H-P, Papp C (2015). Reactivity of graphene-supported Pt nanocluster arrays. ACS Catal.

[93] Podda N, Corva M, Mohamed F, Feng ZJ, Dri C, Dvorak F, Matolin V, Comelli G, Peressi M, Vesselli E (2017). Experimental and theoretical investigation of the restructuring process induced by CO at near ambient pressure: Pt nanoclusters on graphene/Ir(111). ACS Nano.

[94] Franz D, Blanc N, Coraux J, Renaud G, Runte S, Gerber T, Busse C, Michely T, Feibelman PJ, Hejral U, Stierle A (2016). Atomic structure of Pt nanoclusters supported by graphene/Ir(111) and reversible transformation under CO exposure. Phys Rev B.

[95] Pervan P, Lazić P (2017). Adsorbed or intercalated: Na on graphene/Ir(111). Phys Rev Mater.

[96] Mousadakos D, Pivetta M, Brune H, Rusponi S (2017). Sm cluster superlattice on graphene/Ir(111). New J Phys.

[97] N'Diaye AT, Gerber T, Busse C, Mysliveček J, Coraux J, Michely T (2009). A versatile fabrication method for cluster superlattices. New J Phys.

[98] Tillekaratne A, Simonovis JP, Zaera F (2016). Ethylene hydrogenation catalysis on Pt(111) single-crystal surfaces studied by using mass spectrometry and in situ infrared absorption spectroscopy. Surf Sci.

[99] Crampton AS, Rötzer MD, Ridge CJ, Schweinberger FF, Heiz U, Yoon B, Landman U (2016). Structure sensitivity in the nonscalable regime explored via catalysed ethylene hydrogenation on supported platinum nanoclusters. Nat Commun.

[100] Föttinger K, Rupprechter G (2014). In situ spectroscopy of complex surface reactions on supported Pd-Zn, Pd-Ga, and Pd(Pt)-Cu nanoparticles. Acc Chem Res.

[101] Rupprechter G (2021). Operando surface spectroscopy and microscopy during catalytic reactions: from clusters via nanoparticles to meso-scale aggregates. Small.

[102] Garcia C, Truttmann V, Lopez I, Haunold T, Marini C, Rameshan C, Pittenauer E, Kregsamer P, Dobrezberger K, Stöger-Pollach M, Barrabes N, Rupprechter G (2020). Dynamics of Pd dopant atoms inside Au nanoclusters during catalytic CO oxidation. J Phys Chem C.

[103] Bukhtiyarov AV, Panafidin MA, Chetyrin IA, Prosvirin IP, Mashkovsky IS, Smirnova NS, Markov PV, Zubavichus YV, Stakheev AY, Bukhtiyarov VI (2020). Intermetallic Pd-In/HOPG model catalysts: reversible tuning the surface structure by O_2_-induced segregation. Appl Surf Sci.

[104] Panafidin MA, Bukhtiyarov AV, Klyushin AY, Prosvirin IP, Chetyrin IA, Bukhtiyarov VI (2020). Pd–Cu/HOPG and Pd–Ag/HOPG model catalysts in CO and methanol oxidations at submillibar pressures. Kinet Catal.

[105] Mamatkulov M, Yudanov IV, Bukhtiyarov AV, Prosvirin IP, Bukhtiyarov VI, Neyman KM (2018). Pd segregation on the surface of bimetallic PdAu nanoparticles induced by low coverage of adsorbed CO. J Phys Chem C.

[106] Bukhtiyarov AV, Prosvirin IP, Chetyrin IA, Bukhtiyarov VI (2019). Using Sr-XPS to study the preparation features of M-Au/HOPG model catalysts (M = Pd, Ag, Cu). J Struct Chem.

[107] Bäumer M, Freund H-J (1999). Metal deposits on well-ordered oxide films. Prog Surf Sci.

[108] Haunold T, Rameshan C, Bukhtiyarov AV, Rupprechter G (2020). An ultrahigh vacuum-compatible reaction cell for model catalysis under atmospheric pressure flow conditions. Rev Sci Instrum.

[109] Suchorski Y, Rupprechter G (2018). Heterogeneous surfaces as structure and particle size libraries of model catalysts. Catal Lett.

[110] Winkler P, Zeininger J, Suchorski Y, Stöger-Pollach M, Zeller P, Amati M, Gregoratti L, Rupprechter G (2021). How the anisotropy of surface oxide formation influences the transient activity of a surface reaction. Nat Commun.

[111] Zhong J-Q, Wang M, Akter N, Kestell JD, Boscoboinik AM, Kim T, Stacchiola DJ, Lu D, Boscoboinik JA (2017). Immobilization of single argon atoms in nano-cages of two-dimensional zeolite model systems. Nat Commun.

[112] Trentino A, Madsen J, Mittelberger A, Mangler C, Susi T, Mustonen K, Kotakoski J (2021). Atomic-level structural engineering of graphene on a mesoscopic scale. Nano Lett.

[113] Lehtinen O, Kotakoski J, Krasheninnikov AV, Tolvanen A, Nordlund K, Keinonen J (2010). Effects of ion bombardment on a two-dimensional target: atomistic simulations of graphene irradiation. Phys Rev B.

[114] Jablonski A, Zemek J (2009). Overlayer thickness determination by XPS using the multiline approach. Surf Interface Anal.

[115] Henry CR (2005). Morphology of supported nanoparticles. Progr Surf Sci.

[116] Appy D, Lei H, Wang C-Z, Tringides MC, Liu D-J, Evans JW, Thiel PA (2014). Transition metals on the (0001) surface of graphite: Fundamental aspects of adsorption, diffusion, and morphology. Prog Surf Sci.

[117] Stakheev AY, Kustov LM (1999). Effects of the support on the morphology and electronic properties of supported metal clusters: modern concepts and progress in 1990s. Appl Catal A.

[118] Lopez-Salido I, Lim DC, Kim YD (2005). Ag nanoparticles on highly ordered pyrolytic graphite (HOPG) surfaces studied using STM and XPS. Surf Sci.

[119] Cherstiouk OV, Simonov PA, Savinova ER (2003). Model approach to evaluate particle size effects in electrocatalysis: preparation and properties of Pt nanoparticles supported on GC and HOPG. Electrochim Acta.

[120] Hövel H, Becker T, Bettac A, Reihl B, Tschudy M, Williams EJ (1997). Controlled cluster condensation into preformed nanometer-sized pits. J Appl Phys.

[121] Grönbeck H, Barth C (2019). Revealing carbon phenomena at palladium nanoparticles by analyzing the work function. J Phys Chem C.

[122] Rupprechter G, Hayek K, Rendón L, José-Yacamán M (1995). Epitaxially grown model catalyst particles of platinum, rhodium, iridium, palladium and rhenium studied by electron microscopy. Thin Solid Films.

[123] Horiuti I, Polanyi M (1934). Exchange reactions of hydrogen on metallic catalysts. Trans Faraday Soc.

[124] Rupprechter G, Morkel M, Freund H-J, Hirschl R (2004). Sum frequency generation and density functional studies of CO–H interaction and hydrogen bulk dissolution on Pd(111). Surf Sci.

[125] Shaikhutdinov S, Heemeier M, Bäumer M, Lear T, Lennon D, Oldman RJ, Jackson SD, Freund H-J (2001). Structure–reactivity relationships on supported metal model catalysts: adsorption and reaction of ethene and hydrogen on Pd/Al_2_O_3_/NiAl(110). J Catal.

[126] Parker SF, Walker HC, Callear SK, Grunewald E, Petzold T, Wolf D, Mobus K, Adam J, Wieland SD, Jimenez-Ruiz M, Albers PW (2019). The effect of particle size, morphology and support on the formation of palladium hydride in commercial catalysts. Chem Sci.

[127] Chesters MA, Packer KJ, Viner HE, Wright MAP, Lennon D (1996). Variable-temperature, ^1^H NMR study of hydrogen chemisorption on EuroPt-1. J Chem Soc Faraday Trans..

[128] Gruber HL (2002). Chemisorption studies on supported platinum. J Phys Chem.

[129] Guan Z, Xue M, Li Z, Zhang R, Wang B (2020). C_2_H_2_ semi-hydrogenation over the supported Pd and Cu catalysts: The effects of the support types, properties and metal-support interaction on C_2_H_4_ selectivity and activity. Appl Surf Sci.

[130] Schneider W-D, Heyde M, Freund H-J (2018). Charge control in model catalysis: the decisive role of the oxide–nanoparticle interface. Chem Eur J.

[131] Montejo-Alvaro F, Rojas-Chávez H, Román-Doval R, Mtz-Enriquez AI, Cruz-Martínez H, Medina DI (2019). Stability of Pd clusters supported on pristine, B-doped, and defective graphene quantum dots, and their reactivity toward oxygen adsorption: A DFT analysis. Solid State Sci.

[132] Ilari GM, Hage FS, Zhang Y, Rossell MD, Ramasse QM, Niederberger M, Erni R (2015). Carbon-metal interfaces analyzed by aberration-corrected TEM: how copper and nickel nanoparticles interact with MWCNTs. Micron.

[133] Kozlov SM, Aleksandrov HA, Neyman KM (2015). Energetic stability of absorbed H in Pd and Pt nanoparticles in a more realistic environment. J Phys Chem C.

[134] Li J, Guan Q, Wu H, Liu W, Lin Y, Sun Z, Ye X, Zheng X, Pan H, Zhu J, Chen S, Zhang W, Wei S, Lu J (2019). Highly active and stable metal single-atom catalysts achieved by strong electronic metal–support interactions. J Am Chem Soc.

[135] Suchorski Y, Kozlov SM, Bespalov I, Datler M, Vogel D, Budinska Z, Neyman KM, Rupprechter G (2018). The role of metal/oxide interfaces for long-range metal particle activation during CO oxidation. Nat Mater.

[136] Kattel S, Ramírez PJ, Chen JG, Rodriguez JA, Liu P (2017). Active sites for CO_2_ hydrogenation to methanol on Cu/ZnO catalysts. Science.

